# Synthesis of magnetic biochar using *Bauhinia variegata* leaves through one-pot hydrothermal process to degrade malachite green dye by Fenton-like catalysis

**DOI:** 10.1371/journal.pone.0344933

**Published:** 2026-05-14

**Authors:** Ramesh Vinayagam, Gokulakrishnan Murugesan, Sujesh Sudarsan, Thivaharan Varadavenkatesan, Raja Selvaraj

**Affiliations:** 1 Manipal Institute of Technology, Manipal Academy of Higher Education, Manipal, India; 2 Department of Biotechnology, M.S.Ramaiah Institute of Technology, Bengaluru, Karnataka, India; University of Szeged, HUNGARY

## Abstract

This study reports the facile one‑pot hydrothermal synthesis of a magnetite‑embedded magnetic biochar (Bv‑MBC) derived from *Bauhinia variegata* leaves and its application as a heterogeneous Fenton‑like catalyst for malachite green (MG) dye degradation. FESEM revealed a highly porous structure with uniformly distributed spherical iron oxide nanoparticles (mean size: 32.65 nm) embedded within the biochar matrix. BET analysis showed a specific surface area of 10.64 m^2^/g. XRD confirmed the presence of crystalline magnetite with an average crystallite size of 19.91 nm, along with an amorphous carbon signal. VSM measurements gave a saturation magnetization of 30.11 emu/g, sufficient to permit rapid magnetic collection of the catalyst after use. In batch trials, Bv-MBC acted as an efficient Fenton-like catalyst for MG, removing 75.01 ± 1.78% of the dye within 180 min at an initial concentration of 5 mg/L. The kinetic data were described by a second-order model, with the apparent rate constant falling from 0.0034 to 0.0003 L mg^-1^ min^-1^ as the starting MG concentration increased from 5 to 25 mg/L. The synthesized Bv‑MBC exhibited good stability, retaining 85.11% of its initial catalytic activity after five successive reuse cycles. The catalyst showed effective MG removal in multiple real water matrices, indicating potential applicability in complex aqueous environments. Hence, this study highlights a promising and easily separable magnetic catalyst to treat dye-contaminated wastewater.

## 1. Introduction

Driven by rapid industrialization and population growth, the discharge of synthetic dyes from sectors like textiles and pharmaceuticals severely pollutes water bodies, endangering both human and marine life even at low concentrations [1,2]. Malachite green (MG), one of the commonly used textile dyes, is known for its carcinogenic and mutagenic effects. It has a severe impact on aquatic systems, damaging the reproductive and immune systems of aquatic organisms [3]. Traditional wastewater methods, including coagulation, adsorption, precipitation, and membrane separation are inefficient in removing the dye residues in the effluent and hence there is a need for a suitable and efficient process [4]. In this context, Advanced Oxidation Process (AOP) serves as an effective method to break down target contaminants using highly reactive products [5]. Amongst various AOPs, oxidation using Fenton’s reagent has shown its potential to remove various hazardous dyes [6]. However, the traditional homogeneous Fenton process presents several limitations, such as the requirement for acidic pH, the formation of iron sludge, and the use of expensive chemicals. Hence, heterogeneous Fenton-like oxidation processes can suitably address these issues [7].

Iron-based heterogeneous catalysts are reported to be more efficient for dye removal [8], however, these nanoparticles suffer from challenges such as aggregation, reduced activity, and difficult recycling. Therefore, attaching nanoparticles onto porous support is essential for ensuring optimal catalytic activity and facilitating easy separation. Amongst many potential supports, biochar is specifically preferred due to its wide availability and good chemical stability [9]. Hence, iron oxide-impregnated magnetic biochar (MBC) offers notable advantages by preventing nanoparticle aggregation, which further enhances the iron oxide’s catalytic activity. This composite material effectively influences the combined benefits of the iron oxide’s catalytic properties, the ease of magnetic separability, and the stability provided by the biochar support [10].

Some of the plant-based MBC materials that were successfully used to remove methylene blue dye include *Acacia koa* pod covers [11], banana peel [12], and avocado peel [13]. Similarly, MBC from rubber seed shells were used to degrade Rhodamine B [14], whereas coconut-clothed biochar MBC effectively removed methyl orange dye [15]. Building on the porous nature of biochar and the redox activity of iron oxides, this study explores whether magnetic biochar synthesized from leaves of *the Bauhinia variegata* tree, an underutilized biomass commonly known as Orchid Tree or Kachnar, via a one‑pot hydrothermal process, can function as an efficient and reusable heterogeneous Fenton‑like catalyst for malachite green degradation. The *in-situ* incorporation of magnetite nanoparticles is expected to promote effective catalytic activity while enabling easy magnetic recovery and structural stability during wastewater treatment.

## 2. Materials and methods

### 2.1 Preparation of biomass and reagents

The primary raw material used for biochar production was *B. variegata* leaves, collected from the institute premises. To prepare the biomass, the fresh leaves underwent a thorough cleaning process: first washed with tap water to remove surface dirt, followed by rinsing with deionized water to eliminate finer impurities. The cleansed leaves were later oven-dried overnight at 80°C to remove moisture content completely. Post-drying, the leaves were powdered using a grinder and stored at room temperature until needed for synthesis.

All chemicals employed in the experiments were of analytical grade and were used without further purification which included Malachite Green (MG) dye (sourced from HiMedia), FeSO_4_ ⋅ 7H_2_O, NaOH, and hydrogen peroxide (H_2_O_2_) (all sourced from Merck). Deionized water was used exclusively for preparing all aqueous solutions throughout the study.

### 2.2 Synthesis of *Bauhinia variegata*-based Magnetic Biochar (Bv-MBC)

**Fig 1:**
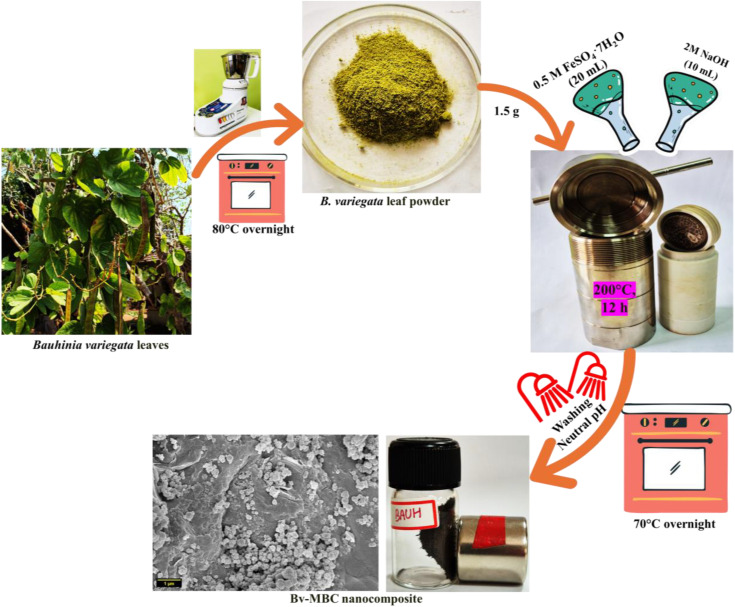
The overall synthesis process of Bv-MBC.

Magnetic biochar derived from *B. variegata* leaves (Bv-MBC) was synthesized using a single-step hydrothermal carbonization technique (Fig 1). In a standard synthesis procedure, 1.5 g of the prepared *B. variegata* leaf powder was added to a solution containing 20 mL of 0.5 M FeSO_4_ ⋅ 7H_2_O and 10 mL of 2M NaOH and mixed for 30 min at room temperature. The resulting suspension was then sealed within a 100 mL hydrothermal reactor. It was heated and kept at 200°C for 12 h to assist the hydrothermal carbonization and incorporation of magnetic iron species. After the reaction period and subsequent cooling to room temperature, the solid contents (Bv-MBC) were collected and washed repeatedly. The washing continued until neutral pH was reached, confirming the removal of any unreacted chemicals or by-products. Finally, the purified Bv-MBC was dried in an oven at 70°C to obtain the final magnetic biochar product.

### 2.3 Characterization studies

A comprehensive set of characterization techniques was applied to evaluate the properties of the synthesized Bv-MBC. Field-emission scanning electron microscopy (FESEM; Neon 40, Carl Zeiss) was used to visualize the surface texture and morphological features, while energy dispersive X-ray spectroscopy (EDX; Oxford Instruments, UK) was employed to determine the constituent elements. Brunauer Emmett Teller (BET) analysis (Smart Instruments, Mumbai, India) was carried out to quantify the specific surface area and pore volume characteristics. X-ray diffraction (XRD; Bruker D8 ADVANCE) was used to identify the crystalline phases, with particular emphasis on the iron oxide species responsible for the magnetic response. Fourier transform infrared (FT-IR) spectroscopy (Cary 660, Agilent Technologies, USA) was used to assign the surface functional groups. X-ray photoelectron spectroscopy (XPS; K Alpha, Thermo Fisher Scientific, UK) provided detailed information on the surface elemental composition and oxidation states. Finally, a vibrating sample magnetometer (VSM; VSM 8600, Lakeshore Cryonics, USA) was employed to record the magnetic hysteresis loop and to determine key magnetic parameters such as saturation magnetization.

### 2.4 Malachite Green (MG) dye degradation experiments

The catalytic performance of the synthesized Bv-MBC toward MG degradation was evaluated in batch mode at 30 ℃. In each experiment, 100 mL of MG solution with an initial concentration between 5 and 25 mg/L was transferred to a conical flask. The reaction was started by adding 1 mL of 30% H_2_O_2_ as oxidant together with 50 mg of Bv-MBC to the dye solution. The flask was kept in a shaking incubator (150 rpm, 30 ℃, 3 h) to maintain continuous mixing and intimate contact among the catalyst, oxidant, and dye during the reaction. At selected time intervals, samples were withdrawn, and the Bv-MBC particles in each aliquot were rapidly separated using a strong neodymium magnet. The clear supernatant was analyzed with a UV-visible spectrophotometer (Shimadzu UV 1900i) at a λ_max_ of 617 nm to determine the residual dye concentration. The removal efficiency was then calculated using Equation (1):


$$\begin{array}{c} \text{Removal efficiency }(\%)=\frac{C_{0}-C_{f}}{C_{0}}\times 100 \end{array}$$ (1)

where $C_{0}$is the initial concentration of MG, and $C_{f}$is the final concentration of MG after the degradation, both expressed in mg/L.

The time profile of MG removal was interpreted using a second-order rate model as given in Equation (2):


$$\begin{array}{c} \left[\frac{1}{C_{f}}-\frac{1}{C_{0}}\right]=kt \end{array}$$ (2)

where “t” denotes degradation time (min), C_0_ and C_f_ denote initial and residual MG dye concentrations (mg/L), and k represents the rate constant (L mg ^−1^ min ^−1^).

### 2.5. Reusability studies

The reusability of Bv-MBC was evaluated under the optimized Fenton-like degradation conditions. In each cycle, 50 mg of Bv-MBC was added to 100 mL of MG solution with an initial concentration of 5 mg/L, followed by the addition of 1 mL of 30% H_2_O_2_. The reaction mixture was maintained at 30 ℃ for 180 min under continuous shaking. At the end of each run, the catalyst was separated by centrifugation at 5000 rpm for 10 min, washed successively with distilled water, ethanol, and again with distilled water to remove any residual dye and reaction intermediates, and then dried for 12 h before being reused in the next cycle. Fresh MG solution and H_2_O_2_ were used for each reuse cycle. The removal efficiency in each cycle was calculated using Equation (1), and the retained catalytic activity relative to the first cycle was used to assess the stability and reuse potential of the synthesized catalyst.

### 2.6. Assessment of MG degradation in real water matrices

The practical applicability of Bv-MBC for Fenton-like degradation of MG was assessed using different real water matrices. Grab samples were collected from well water (WW), tap water (TW), Arabi Falls (AF), Suvarna River (SR), and Manipal Lake (ML), while distilled water (DW) was used as the control matrix. The collected water samples were first filtered to remove coarse suspended impurities and then spiked with MG to obtain an initial concentration of 5 mg/L. For each experiment, 100 mL of the spiked water sample was transferred to a conical flask. To maintain the same operating conditions used in the batch degradation experiments, 50 mg of Bv-MBC and 1 mL of 30% H_2_O_2_ were added to the reaction mixture. The flasks were then kept in a shaking incubator at 30 ℃ for 180 min to ensure continuous contact among the catalyst, oxidant, and dye molecules. The degradation efficiency in each matrix was calculated using Equation (1) to assess the influence of coexisting ions and naturally occurring constituents in real water on the catalytic performance of Bv-MBC.

## 3. Results and discussion

### 3.1. Morphological features and compositional analysis

**Fig 2:**
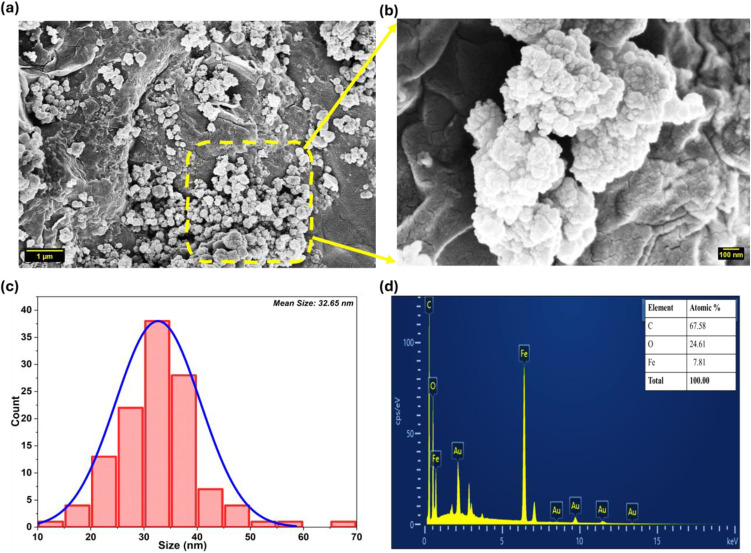
Characterization of Bv-MBC. **(a)** Low-magnification FESEM image (scale bar: 1 µm); **(b)** High-magnification FESEM image (scale bar: 100 nm) of the inset; **(c)** Particle size distribution histogram; **(d)** EDX spectrum with elemental composition.

The low magnification FESEM micrograph (Fig 2a) shows Bv-MBC with a highly porous and irregular surface, where interconnected pores and agglomerated nanoparticles are clearly visible within the biochar matrix, as indicated in the inset. At higher magnification (Fig 2b), these agglomerates are resolved as nearly spherical iron oxide nanoparticles that are evenly dispersed over the biochar surface. Such a porous architecture is characteristic of biochar obtained by hydrothermal treatment, arising from the thermal decomposition and carbonization of the lignocellulosic constituents of *B. variegata* leaves under high-pressure aqueous conditions. This surface morphology is beneficial for environmental applications, because the network of interconnected pores can increase the adsorption capacity of Bv-MBC by providing an extensive accessible surface for MG dye molecules. Comparable porous features have been reported for magnetic biochar derived from rice straw [16] and for materials produced from sewage sludge and woodchips [17].

The particle size distribution of the iron oxide nanoparticles was obtained by image analysis of the FESEM micrographs and is summarized in the histogram in Fig 2c. The nanoparticle diameters ranged from 10 to 70 nm, with a mean value of 32.65 nm. Most particles fell between 20 and 40 nm, which indicates a relatively narrow size distribution. This average size is clearly smaller than that reported for Fe_3_O_4_ nanoparticles in biochar prepared from sludge and woodchips, where values between 50 and 100 nm were observed [17]. Such a size window is favorable for catalytic use, since nanoparticles below 50 nm generally display a high surface-to-volume ratio and therefore provide a greater number of accessible active sites for Fenton-like reactions. The fairly uniform dispersion of Fe_3_O_4_ nanoparticles seen in Fig 2b implies that the single-step hydrothermal route effectively governs nucleation and growth of the magnetic domains and avoids the requirement for a separate impregnation stage. This uniformity helps maintain consistent catalytic behavior by limiting spatial variations in the rate of hydroxyl radical (•OH) generation across the biochar surface, which is crucial for the degradation of MG.

The Bv-MBC material exhibited a mesoporous nature, with a specific surface area of 10.64 m^2^/g, a pore volume of 0.0515 cm^3^/g, and a pore diameter of 19.36 nm, as determined by BET analysis. The pore size is larger than that of the MBC synthesized from avocado peel (8.25 nm) [13], *Egeria najas* (9.8 nm) [18], and peanut shells (16.9 nm) [19]. These properties suggest that Bv-MBC could potentially enhance the concentration of MG dye around active sites during Fenton-type degradation processes.

Figure 2d shows the EDX spectrum and elemental analysis of Bv-MBC. Carbon (67.58%), oxygen (24.61%), and iron (7.81%) are the dominant elements, with only trace levels of phosphorus (P) and gold (Au). The high carbon fraction reflects the biochar matrix derived from *B. variegata* leaves, whereas the substantial oxygen content indicates the presence of surface functionalities rich in oxygen that are characteristic of hydrothermally produced biochar. These groups can enhance MG adsorption through hydrogen bonding and electrostatic interaction and thus act together with the catalytic degradation pathway. The iron fraction confirms that iron oxide nanoparticles are successfully anchored on the biochar and provide the sites required for Fenton-like activity by promoting the formation of •OH radicals from H_2_O_2_. An iron loading of 7.81% is sufficiently high to supply active centers for H_2_O_2_ activation, while low enough to limit iron leaching during operation, an issue often reported for Fenton systems that can cause secondary contamination. The small amount of phosphorus most likely originates from inherent phosphates in *B. variegata* leaves, whereas the gold signal arises from the conductive gold coating applied for FESEM observations.

### 3.2. XRD Studies

**Fig 3:**
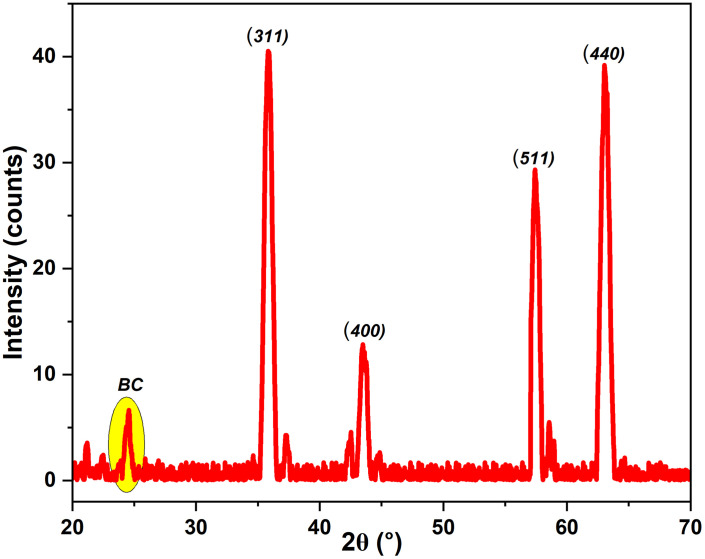
XRD Pattern of Bv-MBC.

The XRD pattern of Bv-MBC (Fig 3) shows sharp and well-defined diffraction peaks that indicate a crystalline phase, together with a small broad hump that reflects an amorphous contribution. The main reflections at 2θ values of 35.85°, 43.51°, 57.48°, and 63.08° can be indexed to the (311), (400), (511), and (440) planes, respectively. These peaks are characteristic of a face-centred cubic spinel ferrite structure corresponding to magnetite [20]. The determined lattice parameter of 8.341 Å is in close agreement with the standard value for magnetite (8.396 Å, JCPDS card number 19–0629) [21]. The slight deviation from the reference value can arise from lattice strain introduced by interaction with the biochar matrix and from the nanoscale nature of crystallites formed *in situ* in Bv-MBC.

A low intensity broad hump centered at about 2θ equal to 24.61°, marked as BC in the figure, is typical of amorphous carbonaceous material [22], and confirms that the *B. variegata* leaves were converted to a biochar component under the hydrothermal conditions. Analysis of the broadening of the intense (311) peak by means of the Scherrer equation gave an average crystallite size of 19.91 nm, indicating that the magnetite phase is present as nanoparticles within Bv-MBC. This nanoscale size is favorable for catalytic use because the high surface area to volume ratio increases the number of accessible active sites. The amorphous biochar fraction provides a porous support with large effective surface area, which promotes adsorption of MG and the uniform dispersion and anchoring of the magnetite nanoparticles formed *in situ*. Hence, XRD results confirm the successful formation of Bv-MBC as a composite produced by the one-pot hydrothermal route. The crystalline magnetite phase supplies the magnetic properties required for easy separation after treatment and serves as the source of catalytically active iron species that enable Fenton-like degradation of MG dye.

### 3.3 FT-IR Studies

**Fig 4:**
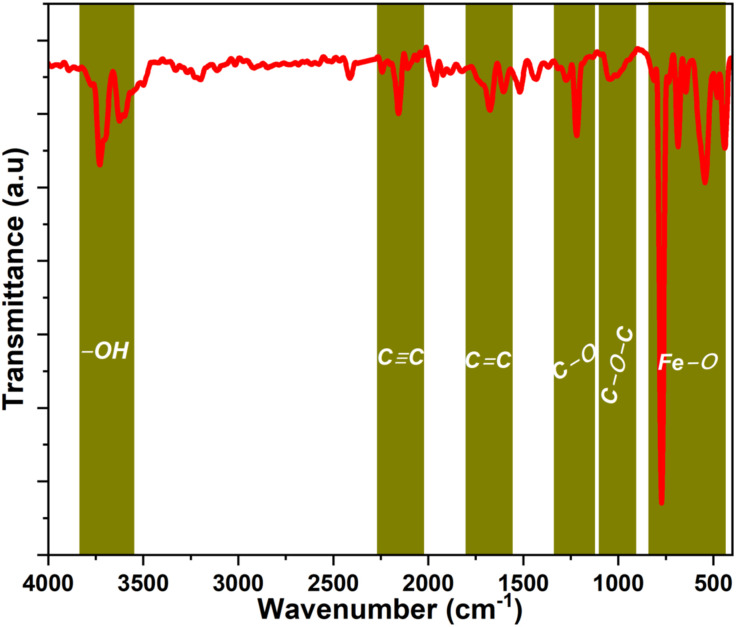
FTIR Spectrum of Bv-MBC.

The FT-IR spectrum of Bv-MBC is shown in Fig 4 and contains several diagnostic absorption bands. Signals near 3730 and 3625 cm^−1^ can be assigned to O–H stretching vibrations of phenolic and alcoholic groups on the biochar surface [23]. The band at 2156 cm^−1^ corresponds to C ≡ C stretching of terminal alkyne groups [24]. A prominent feature at 1678 cm^−1^ arises from aromatic C = C stretching, which can originate from alkenyl domains present in the initial biomass or generated during the hydrothermal treatment [25]. The band around 1220 cm^−1^ is typically associated with C–O stretching, indicating the presence of ether, ester, or phenolic functionalities [26]. The signal at 1040 cm^−1^ is characteristic of C–O–C symmetric stretching in aliphatic ethers and can be related to polysaccharides or alcohol based structures derived from the *B. variegata* leaves [27]. Bands at lower wavenumbers, specifically at 773, 685, and 540 cm^−1^, are attributed to Fe–O stretching vibrations [28], which confirms that iron oxide nanoparticles are successfully incorporated into the biochar framework during the single-step synthesis. These surface functional groups are expected to contribute to MG degradation by supporting adsorption and facilitating the catalytic process.

### 3.4 VSM Analysis

**Fig 5:**
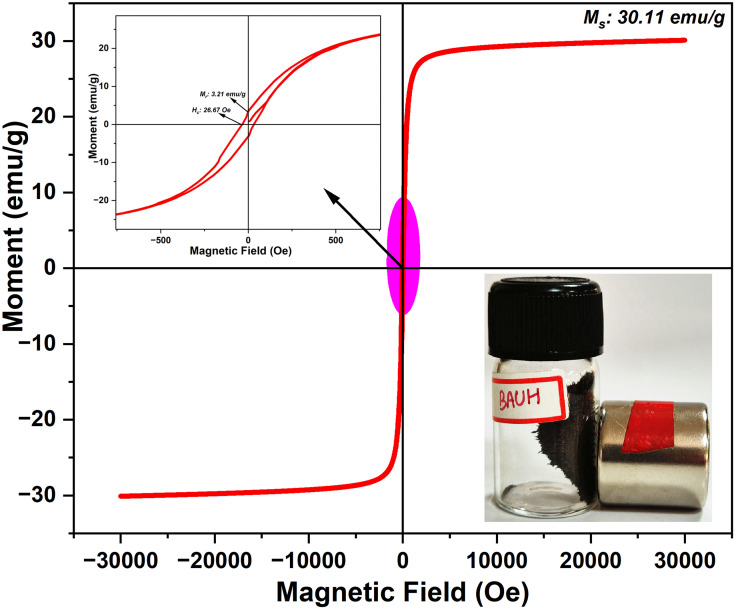
Magnetization Curve of Bv-MBC.

Magnetic behavior of the synthesized Bv-MBC was examined with a vibrating sample magnetometer (VSM) at room temperature to evaluate its suitability for magnetic separation. The hysteresis curve shown in Fig 5 gives a saturation magnetization (M_s_) of 30.11 emu/g. This value represents the maximum magnetic moment attained under a strong external field and confirms that a considerable amount of magnetic iron oxide is incorporated within the biochar matrix. The M_s_ value is substantial enough for practical magnetic separation, as visually demonstrated by the strong attraction of the Bv-MBC powder to a neodymium magnet (as shown in the inset in Fig 5). Bv-MBC exhibited a moderately higher M_s_ value compared to other MBCs reported earlier. For instance, the M_s_ value for MBC from macroalgae is 13.7 emu/g [29], from mangosteen shell is 10.22 emu/g [30], and from *Vateria indica* fruit biomass is 4.74 emu/g [31]. Furthermore, the low coercivity value (H_c_ = 26.67 Oe) observed in the hysteresis loop suggests that the material requires only a weak magnetic field to reverse its magnetization, characteristic of soft magnetic materials. The remanent magnetization (M_r_) of Bv-MBC was 3.21 emu/g. The M_r_/M_s_ ratio, an important parameter for assessing superparamagnetism, was calculated to be 10.67%, which is significantly lower than the widely recognized threshold of 25% for superparamagnetism [32]. This magnetic behavior is advantageous for catalytic use because the particles can be well dispersed in the reaction medium without strong magnetic agglomeration and can still be efficiently collected with an external magnet after the reaction, allowing simple separation and potential reuse.

### 3.5 XPS Analysis of Bv-MBC

**Fig 6:**
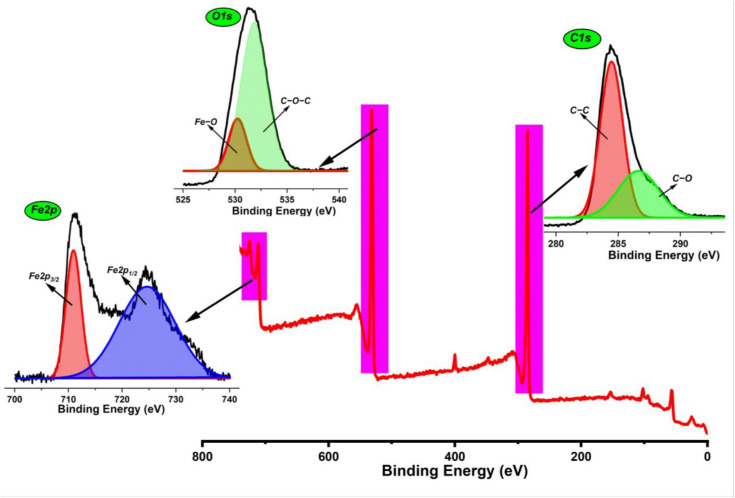
XPS Spectra of Bv-MBC.

Figure 6 presents the XPS survey scan together with the high-resolution spectra of Bv-MBC. The survey spectrum shows iron, carbon, and oxygen as the main surface elements. The high-resolution regions provide insight into their chemical states. In the Fe2p region, two distinct peaks appear at binding energies of 710.95 eV (Fe2p_3/2_) and 724.67 eV (Fe2p_1/2_). These values are typical of magnetite, in which iron occurs in mixed Fe^2+^ and Fe^3+^ states, and they support the formation of magnetite nanoparticles within the biochar [33]. The C1s spectrum can be resolved into contributions from C–C bonds of graphitic or aliphatic carbon at 284.46 eV and from C–O bonds associated with hydroxyl, ether, or ester groups at 286.63 eV [34]. Analysis of the O1s region shows a component at 530.24 eV that corresponds to Fe–O bonds in iron oxide [35] and another at 531.85 eV assigned to C–O and C = O environments, including C–O–C type linkages and other oxygenated functionalities arising from the biochar matrix [36]. These observations confirm that Bv-MBC is a composite material composed of an iron oxide phase integrated with a carbon-rich biochar framework bearing multiple oxygen-containing surface groups, and both parts are expected to contribute to its catalytic behavior.

### 3.6. Degradation Kinetics of MG Dye

**Fig 7:**
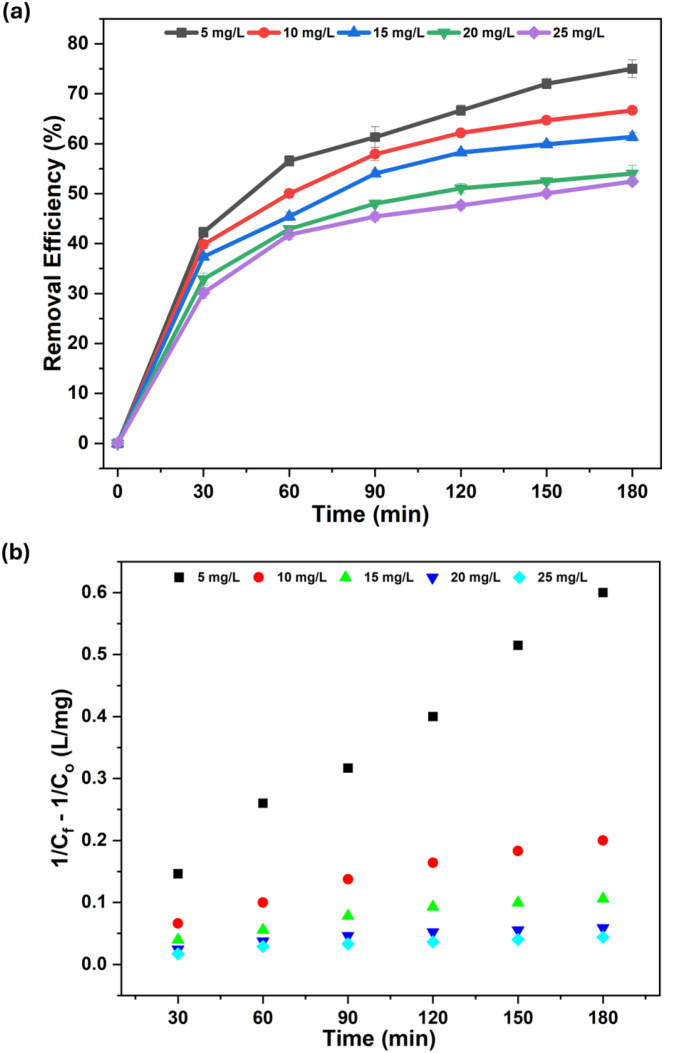
(a) Time-dependent degradation profile of MG dye using Bv-MBC in a Fenton-like system; (b) Second-order kinetic plot for the degradation.

Bv-MBC degraded MG through a Fenton-like pathway and showed a clear time-dependent removal trend (Fig 7a). The gradual fading of colour with time was tracked from the absorption spectra by monitoring the band at 617 nm. The concentration profile in Fig 7a shows a two-stage behavior. A sharp fall in MG concentration occurred during the first 60 min, after which the rate of removal decreased, and the system approached equilibrium at about 180 min. The fast initial stage can be linked to the large number of accessible active sites on Bv-MBC and the abundant formation of hydroxyl radicals immediately after H_2_O_2_ addition [37]. The slower subsequent stage reflects the progressive consumption of H_2_O_2_ and MG as the reaction proceeds.

The initial concentration strongly affected the degradation outcome. As summarized in Table 1, the highest removal of 75.01 ± 1.78% was obtained at the lowest initial MG level of 5 mg/L. When the initial concentration was raised to 25 mg/L, the degradation efficiency dropped to 52.45 ± 0.75%. This decline at higher dye loadings can be corroborated by more intense competition for sorption sites on the catalyst surface and by additional scavenging of •OH radicals by excess MG molecules and reaction intermediates, which together slow the overall degradation [38]. The biochar framework helps to sustain performance by acting as a robust support for Fe_3_O_4_ nanoparticles, limiting their agglomeration and maintaining accessible catalytic sites during the reaction [39].

**Table 1:** Kinetic parameters for MG dye degradation by Bv-MBC.

MG dye concentration (mg/L)	Degradation efficiency (%)	Second-order kinetic constant (L mg^-1^ min^-1^)	R^2^
5	75.01 ± 1.78	0.0034	0.9945
10	66.66 ± 0.49	0.0013	0.9783
15	61.36 ± 0.84	0.0007	0.9684
20	54.01 ± 1.63	0.0004	0.9508
25	52.45 ± 0.75	0.0003	0.9533

Kinetic analysis was used to clarify the degradation pathway. The experimental data fitted well to the second-order kinetic model in Equation (2), with R^2^ values above 0.95 for all initial MG concentrations (Fig 7b and Table 1), indicating that the degradation rate depends on the concentrations of both MG and the oxidizing agent [40]. The second-order rate constant (k) decreased as the initial MG concentration increased, from 0.0034 L/mg/min at 5 mg/L to 0.0003 L/mg/min at 25 mg/L (Table 1). This behavior matches the decline in degradation efficiency at higher initial dye concentrations and confirms the strong influence of reactant concentration on the reaction rate.

The Fenton-like degradation mechanism involves a surface catalytic cycle in which Fe^2+^ sites on Bv-MBC react with H_2_O_2_ to generate highly reactive •OH radicals that oxidize MG into simpler and less harmful products. The Fe^3+^ formed in this step can be reduced back to Fe^2+^ by H_2_O_2_ or through electron transfer from the conductive biochar support [31], which regenerates active sites and sustains the catalytic cycle over the 180 min reaction period. Furthermore, the magnetic character of Bv-MBC enables easy separation of the catalyst with an external magnet and its possible reuse, enhancing the practical applicability of this system for wastewater treatment.

The degradation efficiency discussed here is based on the reduction in MG concentration and visible color removal. While decolorization is a useful indicator of Fenton‑like oxidative activity, it does not necessarily imply complete mineralization of the dye molecules. In heterogeneous Fenton systems, hydroxyl radicals initially cleave chromophoric structures, followed by the gradual oxidation of aromatic intermediates toward low‑molecular‑weight compounds, and eventual mineralization under sufficient reaction severity. Similar behavior has been widely reported for Fenton‑based advanced oxidation processes, where total organic carbon (TOC) removal typically proceeds at a slower rate than color removal [41].

### 3.7. Reusability studies

**Fig 8:**
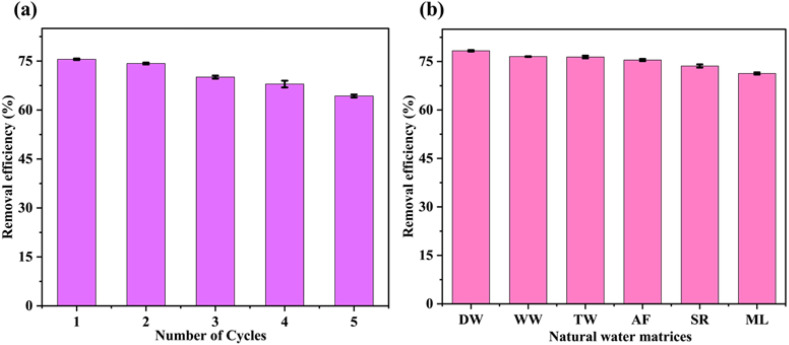
(a) Reusability of Bv-MBC during five successive Fenton-like degradation cycles of MG dye; (b) Degradation performance of Bv-MBC toward MG in different water matrices [distilled water (DW), well water (WW), tap water (TW), Arabi Falls (AF), Suvarna River (SR), and Manipal Lake (ML)].

The reusability of Bv-MBC was evaluated over 5 successive Fenton-like degradation cycles, and the results are presented in Fig 8a. The catalyst showed high degradation efficiency in the first cycle, achieving 75.56% MG removal, and maintained considerable catalytic activity upon repeated use, with removal efficiencies of 74.26, 70.09, 67.96, and 64.31% in the 2^nd^, 3^rd^, 4^th^, and 5^th^ cycles, respectively. The gradual decline in removal efficiency indicates that Bv-MBC retained good catalytic performance even after repeated operation, preserving 85.11% of its initial activity by the 5^th^ cycle. This sustained performance reflects the appreciable structural stability and catalytic durability of the material under repeated Fenton-like conditions. The slight reduction in activity with increasing cycle number may be attributed to the partial blockage of catalytically active sites by adsorbed dye molecules and degradation intermediates [42], as well as minor iron leaching during repeated operation [43], which can reduce the number of surface Fe active sites available for H_2_O_2_ activation.

### 3.8. Assessment of MG degradation in real water matrices

The practical applicability of Bv-MBC for Fenton-like degradation of MG was evaluated in different real water matrices, and the results are presented in Fig 8b. The catalyst exhibited the highest degradation efficiency in DW, achieving 78.32%, which served as the control system with minimal interference from background constituents. In the real water samples, the degradation efficiencies in WW, TW, and AF were 76.53, 76.39, and 75.46%, respectively, indicating that these matrices had only a limited influence on the catalytic process. In contrast, the comparatively lower degradation observed in SR and ML, with removal efficiencies of 73.61 and 71.34%, suggests a relatively stronger matrix effect in these water samples. This reduction may be attributed to a higher content of dissolved organic matter and coexisting inorganic ions [44], which could consume part of the generated reactive species, reduce the effective utilization of H_2_O_2_, and hinder contact between MG molecules and the active sites of Bv-MBC. These results indicate that the Bv-MBC retains good catalytic activity in most real water matrices.

## 4. Conclusion

A magnetically recoverable biochar‑based catalyst (Bv‑MBC) was successfully developed from *Bauhinia variegata* leaves using a simple one‑pot hydrothermal approach, demonstrating the effective valorization of this underutilized biomass. The *in-situ* incorporated magnetite nanoparticles enabled efficient heterogeneous Fenton‑like degradation of malachite green while allowing facile magnetic separation and reuse. The catalyst maintained 85.11% of its initial activity after five successive degradation cycles, indicating good structural stability, with the slight activity loss likely related to partial surface site blockage and minor iron leaching. Bv‑MBC also exhibited consistent performance in various real water matrices, suggesting tolerance toward common matrix constituents such as dissolved organic matter and coexisting ions. In this study, mechanistic interpretation is primarily based on kinetic behavior and surface characterization. Future investigations should focus on detailed mechanistic validation, quantitative leaching analysis, and evaluation under continuous‑flow or large‑scale wastewater treatment conditions. Overall, this work highlights a low‑cost, sustainable, and magnetically separable catalyst with promising potential for dye‑contaminated water remediation.

## Supporting information

S1 Data Minimal data set. (XLSX)
